# Assessing Readiness for Future Maternal Malaria Vaccines: Knowledge, Practices, and Vaccine Attitudes Among Women of Reproductive Age in Malawi

**DOI:** 10.3390/vaccines14040316

**Published:** 2026-03-31

**Authors:** Mandeep Kaur, Flavia D’Alessio, Marion Chirwa Kajombo, Mzati Nkolokosa, Ole F. Olesen

**Affiliations:** 1 European Vaccine Initiative, Im Neuenheimer Feld 515, 69120 Heidelberg, Germany; 2 Department of Language and Communication Studies, Malawi University of Science and Technology, Limbe P.O. Box 5196, Malawi; mckajombo@must.ac.mw; 3 Department of Language and Linguistics, Faculty of Social Sciences, Colchester Campus, University of Essex, Wivenhoe Park, Colchester CO4 3SQ, UK; mn23458@essex.ac.uk

**Keywords:** placental malaria, vaccines, vaccination, maternal immunisation, Malawi, vaccine hesitancy

## Abstract

Background: Placental malaria (PM) is a serious complication of malaria in pregnancy (MiP). It has major repercussions for mothers’ and neonates’ health, particularly in sub-Saharan Africa (SSA). As current preventive measures lose efficacy due to drug resistance, malaria vaccines can play a crucial role in malaria control. The main objective of this study was to generate evidence that can guide the design of social and behaviour change interventions to raise awareness of PM and improve vaccine acceptance. Methods: A facility-based cross-sectional survey was conducted; five dichotomised indicators were constructed; multivariate logistic regression was adjusted for age, education, and districts; and prespecified sensitivity analyses were done. Results: General malaria knowledge and preventive practices were high. Many women (53.4%) reported having had experienced fever during pregnancy. Prevention behaviour was not significantly associated with age or education. Both high knowledge (aOR 0.30, 95% CI 0.16–0.57) and perceived risk awareness (aOR 0.35, 95% CI 0.18–0.68) were lower for Mpemba than for Thyolo. Biomedical healthcare services were less likely utilised by women in Madziabango as compared to Thyolo (aOR 0.47, 95% CI 0.23–0.96). Although 92% acknowledged possible harm, nearly all of them (97%) reported willingness to accept a future maternal malaria vaccine. Conclusions: There was a high level of maternal malaria vaccine acceptability; however, these findings suggest that local context-specific delivery strategies could be useful for effective future PM vaccine introduction.

## 1. Introduction

Malaria continues to pose a serious threat to global health and contributes significantly to the disease burden in sub-Saharan Africa (SSA). In 2024, there were approximately about 610,000 malaria-related deaths and 282 million cases of malaria, and 13 million pregnant women were infected with malaria in the African region [1]. Placental malaria (PM) occurs with the attachment of *Plasmodium falciparum*-infected red blood cells to the placenta through the VAR2CSA protein. This adhesion triggers inflammation, which then reduces placental blood flow, as well as limiting the nutrient and oxygen transfers to the foetus, potentially leading to problems such as maternal anaemia, low birth weight, and premature birth [2,3]. Nearly 17% of pregnancies are affected by placental malaria globally, and this number rises up to 25% in SSA, causing an estimated 75,000–200,000 infant deaths annually [4]. Women are at higher risk during their primary and secondary pregnancies because they have not built up enough immunity to VAR2CSA-associated parasites [2]. In 2024, the majority of malaria cases (94%) and deaths (95%) occurred in the African region [1].

Malaria is endemic in Malawi, with 2021 figures suggesting roughly 4.4 million cases and 7392 deaths [5]. National malaria control efforts in Malawi have achieved some success [6]; however, the emergence of drug resistance, together with low IPTp3 coverage (56%) [7], presents major obstacles to malaria in pregnancy (MiP) prevention and reduction of harmful outcomes of PM [8]. These limitations emphasise the necessity of alternative interventions, such as vaccines, and there have been recent developments in the progress of malaria vaccines [9,10,11]. 

It is essential to understand the perceptions, practices, knowledge, and beliefs of mothers and women of reproductive age to encourage the uptake of potential vaccines. These factors notably influence vaccine decisions and shape the perceptions of future mothers toward disease and the vaccine [12]. National surveys and behaviour studies show persistent gaps in knowledge and prevention behaviour among women of reproductive age. The 2021 Malawi Malaria Indicator Survey (MMIS) shows a significant malaria burden and documents behavioural and psychosocial barriers that continue to impede optimal malaria practices among vulnerable populations [7,13].

Surveys are common tools to gather essential data, thereby informing the design of future interventions aimed at promoting community involvement, acceptance and adherence [14]. While general malaria knowledge among Malawian women has been described in national and peer-reviewed surveys, robust, recent evidence specifically on women’s knowledge of malaria in pregnancy, especially among rural populations, remains sparse [15,16,17,18,19]. The main objective of this study was to generate evidence that can guide the design of social and behaviour change interventions to raise awareness of PM and improve their vaccine acceptance.

## 2. Materials and Methods

### 2.1. Study Area, Design, and Population

Data was collected through a community-based cross-sectional survey in three health facilities and their catchment areas in the Blantyre (Mpemba, Madziabango) and Thyolo districts of southern Malawi from May to July 2024. (A catchment area refers to the geographic area and population that the facility primarily serves.) The study sites were selected in collaboration with the partner institution based on accessibility and logistical feasibility, as well as their relevance to the study objectives, including relatively high malaria burden and teenage pregnancy rates. A target sample size of approximately 300 participants (around 100 per site) was defined pragmatically to allow for comparisons across the three districts within the available study period. The study regions comprised a mix of rural and semi-urban populations, with the majority of the participants belonging to the Mang’anja and Lhomwe ethnic groups, primarily following Christianity. Eligible participants were women of reproductive age with at least one pregnancy, who were able and willing to provide informed consent. The reproductive age range in Malawi is typically defined as 15 to 49 years for women [17]. Although village-based sampling was originally planned, participants were recruited from antenatal care (ANC) facilities. The participants were pregnant women who visited ANC units at the District Health Centres of Thyolo, Mpemba, and Madziabango. They came from multiple villages within each district, with variable numbers of respondents per village. Although patients generally seek care at their own district’s facility, some pregnant women may also attend health facilities in another district depending on distance, preference, or referral. 

A structured questionnaire was developed in English and subsequently translated and carried out in Chichewa. The questionnaire was developed through (a) review of relevant peer-reviewed literature and existing maternal vaccine acceptability instruments such as knowledge, attitude, practices (KAPs) and (b) informed by hesitancy and established measurement frameworks and question banks (WHO BeSD tools; WHO SAGE vaccine hesitancy determinants and sample questions; validated acceptance constructs such as the 5C scale) [20,21]. The survey included 24 questions organized into the following topics: (i) socio-demographic characteristics, (ii) knowledge of malaria, (iii) malaria prevention practices, (iv) risk perception, (v) healthcare-seeking practices for malaria, and (vi) vaccine attitude. Data was collected at the District Health Centres by trained research assistants; women were interviewed in the absence of healthcare workers in the room. The study team was trained in the study protocol, the questionnaire, and the consent process.

#### The Local Understanding of Placental Malaria

**Figure 1:**
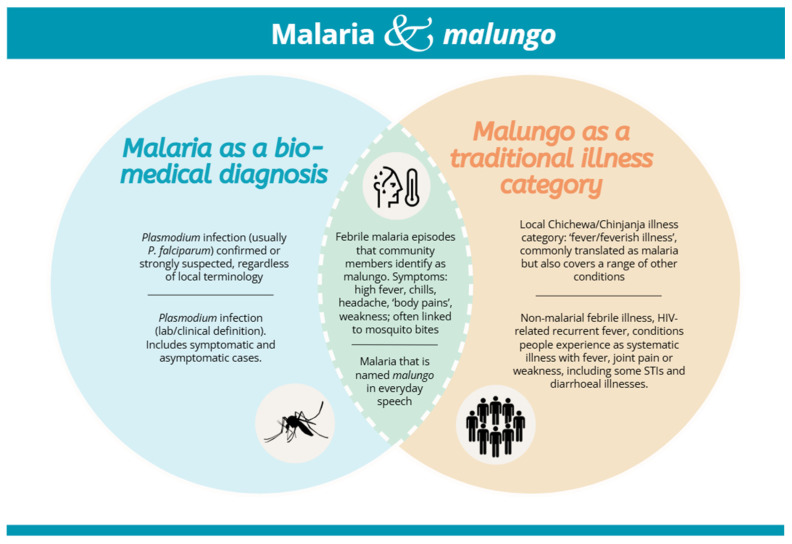
Conceptual relationship between the illness category *malungo* and biomedical malaria in local languages in Malawi.

The linguistic shift from English to Chichewa has implications for the interpretation of key terms used in the interviews, including the use and meaning of “*malungo*”, a term often used in the literature to refer to malaria [15,16,22]. In Malawi, the term ‘*malungo*’ is used to describe fever and related illnesses, and it does not carry the same precision as ‘malaria’ caused by *Plasmodium falciparum* (see Figure 1). Previous studies showed that “*malungo*” is a broad, culturally infused term utilised to denote several ailments: People use it for a range of febrile conditions, diarrhoea, sexually transmitted infections and Acquired immunodeficiency Syndrome (AIDS), not just malaria. Hence, the general use of ‘*malungo*’ can obscure distinctions between malaria, flu and other illnesses [15,22]. Some communities in Malawi do not use or recognise the term *malungo* at all [23]. Based on ethnographic KAP studies in Malawi, *malungo* is the dominant vernacular term used to translate ‘malaria’. This implies that most lay-recognised malaria episodes are classified as *malungo*, but not all illnesses called *malungo* correspond to malaria in biomedical terms [15,22,24,25,26,27].

In our study, participants used the term ‘*malungo*’ for a variety of illnesses, e.g., general fever, along with body pains, shaking in the knees, shivering, and joint pain, but understood that only the hospital can confirm if it is indeed “*malungo*”, the equivalent of malaria.

Participants did not mention any specific *malungo* related to *nsengwa* (placenta). The term ‘placental malaria’ lacks direct translation in local languages. Combining the words “*nsengwa*” and “*malungo*” lacks cultural and linguistic coherence because they are not recognised as established or meaningful expressions in local vernacular usage.

To bridge this conceptual gap, we initially developed a local term: *Malungo a mu nsengwa ya amayi oyembekezera* (“fever of the placenta”). When asked about their knowledge of PM using these terms, participants mentioned that *nsengwa* (placenta) itself cannot be infected by *malungo*; rather, it is the mother who suffers from *malungo*. Therefore, ‘*malungo* in pregnancy’ is the term chosen to be used rather than ‘malaria in pregnancy’ to conduct this study, based on their experience and context. Because the term “placental malaria” does not have a vernacular equivalent and was not seen as a separate illness, questions about vaccine acceptability were asked in terms of desire to get a “*malungo*-in-pregnancy” vaccine. The term “placental malaria” is used in this paper to describe the intended indication in a biomedical way, rather than as a locally known illness category.

While not identified explicitly as placental malaria, these accounts nevertheless offer helpful information regarding women’s experiences and their community’s perception, knowledge, attitudes, and practices concerning *malungo* in pregnancy, which may reflect women’s lived experiences of conditions consistent with placental malaria. As community usage does not distinguish ‘*malungo*’ (umbrella term for febrile illnesses) from biomedical ‘malaria’, we used ‘*malungo*’ throughout this paper.

### 2.2. Data Analysis

All data were initially processed in Microsoft Excel for coding and cleaning and subsequently analysed in R (version 4.5). Descriptive statistics were generated for all variables.

**Figure 2:**
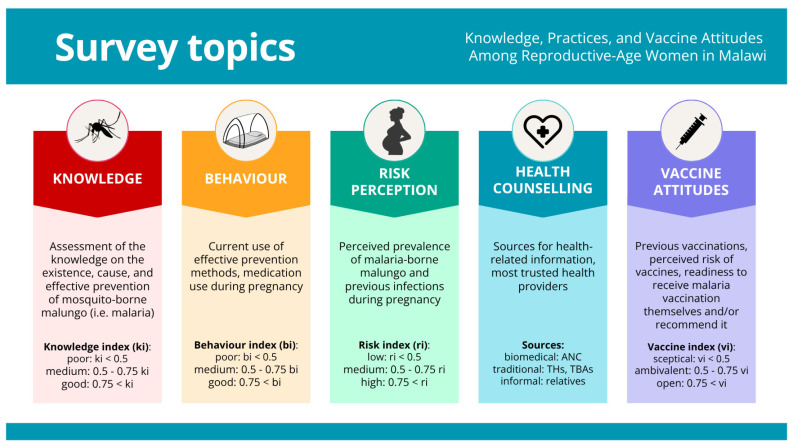
Key thematic areas explored in the survey on knowledge, behaviour, risk perception, health counselling and vaccine attitudes among reproductive-age women in Malawi.

Five outcome domains were established using the questionnaire (see Figure 2), which included the following: the *knowledge index* (ki, 4 items), the *prevention behavioural index* (bi, 3 items), the *risk awareness index* (ri, 3 items), the *health counselling source* (hc, 4 items), and the *vaccine attitude index* (vi, 6 questions) (see Table S2). Item responses were coded numerically, and index scores were calculated as the mean of item responses for each participant, yielding continuous values from 0 to 1. If one item within an index was missing, the index was calculated from the remaining items; if more than one item was missing, the index was not calculated for that respondent. Full item coding and rules are provided in Table S2.

Except for the risk index, higher index values indicated knowledge, behaviours, health counselling, and vaccine attitudes consistent with biomedical understandings of malaria, while lower values reflected responses less aligned with these frameworks. Higher values for the risk index indicated a higher perceived risk of transmission in the community, and/or personal past malaria infections in pregnancy.

For regression analyses, indices were dichotomised into high versus low values using outcome-specific thresholds based on their distributions (e.g., median split for most indices; predefined threshold ≥0.8 for vaccine attitude). Health counselling responses were categorised and dichotomised into “exclusively biomedical” versus “any informal/mixed” sources.

Bivariate associations between categorical variables were assessed using Fisher’s exact test or Pearson’s chi-square test, as appropriate. Multivariable logistic regression models were fitted to estimate adjusted associations between sociodemographic factors and high index values, including age group (<25 vs. ≥25 years), education level (lower vs. higher), and district as covariates. Results are reported as adjusted odds ratios (aORs) with 95% confidence intervals (CIs).

## 3. Results

### 3.1. Participants’ Demographic Characteristics

**Figure 3:**
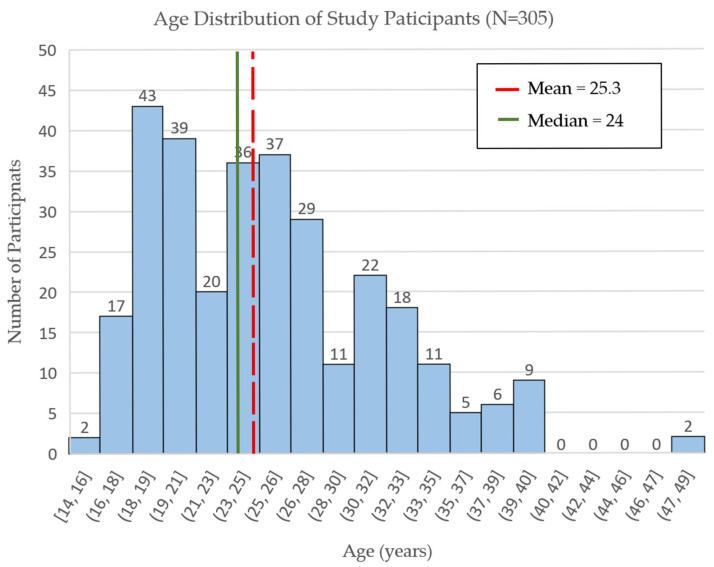
Age distribution of study participants.

A total of 307 participants, with a mean age of 25.3 years (median age 24, interquartile range 20–29), participated in the survey (see Table 1). The targeted reproductive age range was 15–49 (see Figure 3). One participant outside the age range was excluded from the dataset. Another participant who only answered questions on demographic data and left all other questions unanswered was also excluded. This resulted in a final dataset of 305 participants.

Most participants, 62.6% (*n* = 191), had only attended primary education, 31.8% (*n* = 97) had secondary education, and one participant held a tertiary education diploma. Most participants were in their 20s (56.4%, *n* = 172), and the median age of the group was 24. Adolescents younger than 20 made up 19.7% (*n* = 60) of participants, with a similar number of older women in their 30s (22%, *n* = 67). A small minority was in their 40s (2%, *n* = 6). Participants came from the districts of Mpemba (34.8, *n* = 106), Madziabango (32.1%, *n* = 98), and Thyolo (33.1%, *n* = 101). Within districts, participants from Thyolo came from 50 different villages (for seven participants no village was recorded), those from Mpemba came from 54 different villages (village information missing for 3), and those from Madziabango came from 34 villages (with village information missing for 14), with similar participant numbers from each. Two women from villages within Thyolo were surveyed at the Madziabango health care facility, and were subsequently grouped with other women from Thyolo instead of the Madziabango group.

### 3.2. Knowledge About Malungo, Perceived Causes and Prevention

Out of 305 participants, 98% (*n* = 299) reported having heard about a disease called malungo. Regarding perceived causes of malungo, 62.3% (*n* = 190) mentioned mosquitoes as the sole cause, while 9.5% (*n* = 29) mentioned mosquitoes along with other explanations. A total of 10.5% (*n* = 32) of participants attributed malungo to not following prevention methods or to environmental factors, without mentioning mosquitoes, such as not sleeping under bed nets, the presence of stagnant water, or unclean surroundings. A total of 0.7% (*n* = 2) of participants provided symptom-based explanations only, such as shivering, coldness, fever, cough, or flu. Notably, 12.1% (*n* = 37) reported that they did not know the cause, while 4.9% (*n* = 15) provided unscorable responses (stating “yes” without further explanation).

To understand whether respondents conceptualized malungo predominantly as the biomedical disease malaria, or under the broader category of malungo (which encompasses other febrile diseases as well), answers were categorized based on whether they belonged exclusively to malaria, to malungo (general hygiene, flu-like symptoms), or to a mix of both. The majority (79.0%, *n* = 241) described malungo causes consistent with biomedical malaria only, by either mentioning mosquitoes and/or the *plasmodium* parasites or by mentioning not following malaria prevention methods (“not sleeping under a bed net”, “keeping stagnant water”, “not taking antimalarial drugs”). A smaller proportion (3.3%, *n* = 10) provided mixed explanations that combined malaria-related responses and alternative environmental or symptom-based interpretations, which are aligned to the broader illness category of malungo. Very few respondents (0.7%, *n* = 2) attributed malungo exclusively to causes unconnected to malaria, such as hygiene, coldness, or flu-like symptoms.

#### Knowledge Index

A composite malaria knowledge index was calculated from the “Knowledge Index” dataset. The index was dichotomized into high versus low knowledge using the sample median as the cutoff. Most respondents demonstrated at least moderate understanding of malaria, as the sample median was a knowledge index of 0.69 (with a mean of 0.72). Education was significantly associated with higher malaria knowledge (aOR 1.83, 95% CI 1.09–3.11, *p* = 0.024). Age was not associated with knowledge level (aOR 0.87, 95% CI 0.54–1.40, *p* = 0.561). Knowledge level differences particular to each district were statistically significant. No significant difference was observed between Madziabango and Thyolo (aOR 0.85, 95% CI 0.48–1.50, *p* = 0.572). In contrast, respondents from Mpemba had substantially lower odds of high knowledge compared to both Thyolo (aOR 0.23, 95% CI 0.12–0.41, *p* < 0.001) and Madziabango (aOR 0.27, 95% CI 0.14–0.49, *p* < 0.001) (see Table 2).

### 3.3. Prevention Behaviour

The majority of study participants (78.5%; *n* = 241)—see Table 3—mentioned sleeping under a mosquito net as a preventive method. Among these, 53.1% (*n* = 162) mentioned only a mosquito net (Madziabango: 37 responses (38%), Mpemba: 56 responses (53%), Thyolo: 71 responses (70%).), while others combined this with other practices such as filling the stagnant water bodies (6.2%; *n* = 19), clearing the surroundings (5.6%; *n* = 17), burning coil/Doom (Doom is the brand name of the local mosquito repellent spray) 6.2% (*n* = 19), and taking antimalarial drugs 2.0% (*n* = 6). Additionally, 12.7% (*n* = 39) of participants included eating clean or hygienic food, burning animal dung, using mosquito repellents, and burning bushes or herbs as localised practices to repel mosquitoes. Around 7.8% (*n* = 24) said ‘no’, indicating that they do not know how to prevent *malungo*, and 5.5% (17) said ‘yes’, without specifying a method. While the majority associate mosquito nets with prevention, only 6 of 305 participants mentioned taking antimalarial drugs as prevention methods. Fifty-four participants said they sleep under mosquito net and are currently taking antimalarials; however, pregnancy status at the time of the interview was not systematically recorded, and therefore these answers cannot be interpreted as pregnancy-specific prevention behaviours.

Most women 82.1% (*n* = 252), said they would take medication if they contracted *malungo* in pregnancy. A minority answered either “I do not know” 6.2%, (*n* = 19) or “no” 3.6%, (*n* = 11), citing reasons such as not being aware, fear of harm to the baby or lack of money.

The composite behaviour index was generally high, with a mean of 0.86 and a median of 0.92, reflecting the widespread use of bed nets and willingness to take medication to treat malaria. Multivariable logistic regression adjusted for education, age, and district showed that education level was not associated with above average preventive behaviour (aOR = 1.57, 95% CI 0.88–2.81, *p* = 0.126); see Table 4. Age was likewise not associated with preventive behaviour, with women aged ≥25 years showing similar odds of good preventive behaviour compared with those aged <25 years (aOR = 1.06, 95% CI 0.61–1.85, *p* = 0.831).

In contrast, the district was a significant predictor. Compared with Thyolo, women from Madziabango had substantially lower odds of above-average preventive behaviour (aOR = 0.26, 95% CI 0.13–0.50, *p* < 0.001), and women from Mpemba also had substantially lower odds (aOR = 0.26, 95% CI 0.13–0.49, *p* < 0.001). There was no difference between Mpemba and Madziabango (aOR = 1.00, 95% CI 0.47–2.17, *p* = 0.997). Overall, these findings indicate that preventive behaviour differed by district, with Thyolo showing stronger behavioural index scores than both Madziabango and Mpemba, while education and age were not significant predictors in the adjusted model.

### 3.4. Perceived Risk Awareness

Perceived malaria risk awareness was moderate overall. Two-thirds of respondents, 63.9%, (*n* = 195) believed their area was high-risk for *malungo*, while 28.2%, (*n* = 86) perceived it as low risk, 5.6% said they did not know (*n* = 17), while data was missing for 2.3% (*n* = 7). To the question “Did you have *malungo* in this/your last pregnancy?”, 68.5%, (*n* = 209) said ‘No’, 25.6%, (*n* = 78) said ‘Yes’, while data was missing for 5.9% (*n* = 18). To the question “Did you have malaria in any other previous pregnancy?”, 61.0% (*n* = 186) said ‘No’, 38.7%, (*n* = 118) said ‘Yes’, while data was missing for one person (0.3%).

The mean of the risk index was 0.46, with a median of 0.33 (a higher index indicating more perceived risk/experiences of infection), indicating low-to-medium perceived risk of malaria infection. Neither education nor age was significantly associated with the risk index (see Table 5). Women with higher education had similar odds of high risk index compared to those with lower education (aOR = 0.94, 95% CI 0.56–1.57, *p* = 0.817). Likewise, women aged ≥25 years had slightly higher odds of a higher risk index than younger women, but this difference was not statistically significant (aOR = 1.12, 95% CI 0.70–1.79, *p* = 0.643).

In contrast, the risk index differed by district. No significant difference was observed between Madziabango and Thyolo (aOR = 0.81, 95% CI 0.46–1.42, *p* = 0.455). However, women in Mpemba had significantly lower odds of a high-risk index compared to both Thyolo (aOR = 0.31, 95% CI 0.17–0.56, *p* < 0.001) and Madziabango (aOR = 0.39, 95% CI 0.21–0.69, *p* = 0.002).

### 3.5. Health Counselling

**Figure 4:**
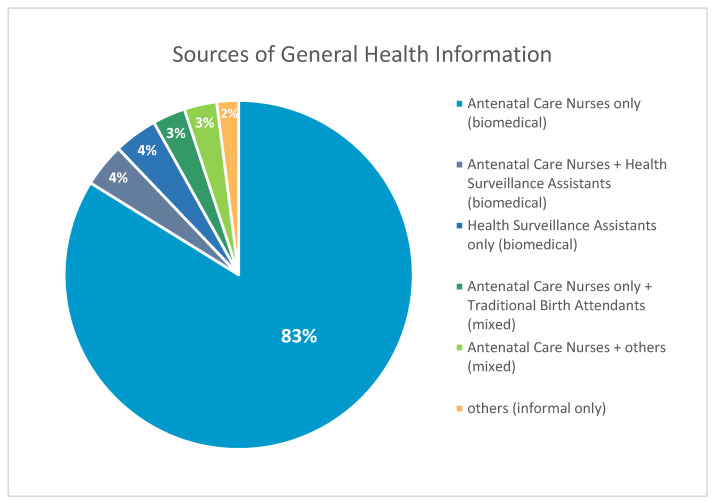
Sources of general health information among study participants.

Most participants (83%; *n* = 255) stated ANC nurses as sole healthcare advisors. The participants showed strong preference for formal healthcare and trust in hospitals and clinics. They described biomedical care as routine, safe, and professional. This was reflected in statements such as: “Hospitals are reliable,” “We trust our hospitals,” and “They have all required resources.” (see Figure 4). However, others mentioned other medical providers such as HSAs 4.2%, (*n* = 13) and non-biomedical and informal sources of health counselling such as TBAs 3.2%, (*n* = 10), relatives 1.9%, (*n* = 6), village chief, relatives, parents and care group, newspapers, community gatherings, and friends 1.6%, (*n* = 5). Few stated only HSAs 4.2%, (*n* = 13) as their medical advisors, or only relatives 0.97%, (*n* = 3). No data was provided for 0.65% (*n* = 2) participants.

Responses to whether women visited TBAs and/or THs varied and often included explanations. Only 4.5% (*n* = 14) said they used TBAs/THs services. Among them, three participants reported using TBAs services to get *Mulimbiko*, a traditional herbal medicine made from *Hippocratea parviflora* leaves, to avoid miscarriage during pregnancy [28]. The survey answers revealed significant scepticism towards TBAs and traditional healers. Common concerns mentioned were fear of miscarriage, “some TBAs deceive”, “they are after money”, “they lie”, “do not have equipment”, “they do not have expertise in medicine”, “they are not permitted to operate”.

To understand whether women who preferred exclusively biomedical counselling differed from those who also accessed informal health counselling, education, age, and district association were analysed between women with a health counselling index of 1 (exclusively biomedical), and those <1 (not exclusively biomedical). Education level showed no association with counselling preference (see Table 6). Women with higher education had similar odds of relying exclusively on biomedical counselling compared to those with lower education (aOR = 0.98, 95% CI 0.55–1.78, *p* = 0.958). In contrast, age was a significant predictor. Women aged ≥25 years were more than twice as likely to rely exclusively on biomedical counselling compared to younger women (aOR = 2.10, 95% CI 1.22–3.69, *p* = 0.009).

Health counselling behaviour also differed by district. Compared with Thyolo, women in Madziabango had significantly lower odds of relying exclusively on biomedical counselling (aOR = 0.40, 95% CI 0.21–0.76, *p* = 0.006), while no significant difference was observed between Mpemba and Thyolo (aOR = 1.14, 95% CI 0.56–2.33, *p* = 0.716). Women in Mpemba were also significantly more likely to rely exclusively on biomedical counselling than those in Madziabango (aOR = 2.84, 95% CI 1.49–5.58, *p* = 0.002).

### 3.6. Attitude Toward Vaccination

There was a high rate of vaccination coverage and willingness among the participants. The majority of women (82.4%; *n* = 253) reported getting a vaccine in the past five years. The majority (83.4%; *n* = 256) also reported receiving a tetanus vaccine recommendation during pregnancy. A vast majority of women (92.8%; *n* = 283) reported having been vaccinated against tetanus in their current pregnancy, while 6.2% (*n* = 19) had not (missing data for 1%, *n* = 3).

Despite this, safety concerns regarding vaccines were prevalent. A majority of women (92.1%; *n* = 281) endorsed the statement that vaccines “may carry a possibility of harm for the baby”, and only 4.9% (*n* = 15) believed there was no harm (missing data for 1%, *n* = 3). Despite this, participants were still open towards receiving vaccinations: Willingness to accept a future malaria vaccine recommended for pregnant women was very high (97.4%; *n* = 297). Similarly, many respondents said they would advise their daughters to get vaccinated (94.4%, *n* = 288). Some participants were not willing to accept a future vaccine (2.0%, *n* = 6) or recommend it to their daughters (4.6%, *n* = 14). A small amount of participants were uncertain about receiving a vaccine themselves (0.7%, *n* = 2) or recommending it to their daughters (0.3%, *n* = 1). Data was missing from two participants (0.7%) on whether they would recommend it to their daughter. This reveals a notable paradox: respondents in the small minority who believed that vaccines were generally safe more often considered to senot vaccinate their children after birth (60%), compared to respondents who did believe in the potential harm of vaccines (2.6%). However, this is due to the large number of participants who were concerned about vaccine safety and who chose not to answer the question of whether they would vaccinate their baby after birth (73.9%). This could indicate either indecision or fear of potential stigma in answering “no”, or high compliance and willingness despite persistent concerns about vaccine safety (see Table 7).

Within the subset analysis, the participants who expressed greater concern about the potential harm of vaccines, appeared to be generally better informed about the existence of malaria vaccines. Just over half of participants (57.4%, *n* = 175) had heard of malaria vaccines, but many had not (42.4%, *n* = 129; missing data 0.3%, *n* = 1). Of those who were not worried about harmful effects of vaccines, almost three quarters 73.3% (*n* = 255) had not heard of malaria vaccines for children yet as it first became available for children in Malawi in April 2019. However, the number of respondents who were not concerned about vaccine safety was in general quite small. It is also possible, that the participants who expressed greater concern about vaccine safety had been more exposed to information about vaccines than those who were less concerned, with both positive and negative messages about vaccine safety.

Interestingly, participants who expressed greater concern about vaccine safety were somewhat more likely than those who were less concerned to report willingness to receive the vaccine themselves (97.2% vs. 86.7%) as well as to recommend it to their daughter (95.4% vs. 80%). Among women who were willing to get vaccination for themselves, nearly all (96.0%, 286 of 298) would advise it to their daughters, while a small minority (3.3%, 10 of 298) would not recommend it. Among the very small number of respondents who would reject a vaccine themselves (2.0%, *n* = 6), half would still recommend it to their daughter.

#### Vaccine Index

To assess factors associated with more positive vaccine attitudes, multivariable logistic regression was used to compare women with a vaccine attitude index of ≥0.8 (median: 0.8; mean: 0.75) to those with lower scores. Education was not significantly associated with vaccine attitude (see Table 8). Women with higher education had slightly higher odds of a more positive vaccine attitude than those with lower education, but this difference was not statistically significant (aOR = 1.23, 95% CI 0.70–2.19, *p* = 0.482). Age was also not significantly associated with vaccine attitude, although women aged ≥25 years had somewhat higher odds of a more positive vaccine attitude than younger women (aOR = 1.47, 95% CI 0.88–2.50, *p* = 0.146).

District-level differences were modest and did not reach statistical significance. Compared with Thyolo, women in Madziabango had lower odds of a vaccine attitude index ≥ 0.8, with borderline evidence of a difference (aOR = 0.53, 95% CI 0.28–1.01, *p* = 0.057), while women in Mpemba did not differ significantly from those in Thyolo (aOR = 0.79, 95% CI 0.41–1.53, *p* = 0.495). There was also no significant difference between Mpemba and Madziabango (aOR = 1.49, 95% CI 0.80–2.77, *p* = 0.209). Overall, the analysis did not identify strong demographic or geographic predictors of more positive vaccine attitudes in this sample.

## 4. Limitations

The participants were recruited from antenatal care (ANC) facilities in three districts in southern Malawi, rather than through community-based sampling. The sample therefore reflects women engaged with formal healthcare services and may underrepresent those with limited or no facility access, limiting generalizability to other settings.

The data were collected through interviewer-administered questionnaires within health facilities. Despite the absence of healthcare staff during interviews, responses may have been influenced by social desirability, particularly for preventive behaviours and vaccine attitudes. Pregnancy status at the time of interview and detailed reproductive history (e.g., gravidity/parity) were not systematically recorded, and although participants were recruited from ANC clinics and were thus likely pregnant, this could not be formally verified or analysed.

Several variables relied on self-reported past experiences, including malaria in previous pregnancies, and are therefore subject to recall bias. In addition, some items, particularly those related to infant vaccination intentions, had substantial missing data, which may have affected the robustness of those findings.

Village representation was uneven across districts, with some villages contributing disproportionately more respondents than others. This was most pronounced in Madziabango, where a single village accounted for around one-fifth of participants, while in Thyolo and Mpemba, a small number of villages also contributed multiple respondents alongside many villages represented by only one participant. No adjustment for clustering at village level was performed, and this uneven distribution may have influenced the precision of estimated associations.

## 5. Discussion

Overall, most participants showed high awareness of *malungo* and its prevention methods. This aligns with national trends in Malawi [7], often attributed to longstanding public health campaigns [18,29]. However, we found consistent disparities among districts regarding perceived risk awareness, trust in HCWs counselling, and vaccine confidence. Although general awareness of *malungo* was high, specific awareness about “*Malungo a mu nsengwa ya amayi*,” (placental malaria) was significantly lower. This knowledge gap is striking, given the major role of PM in maternal anaemia, stillbirth, and low birth weight [2]. Bridging biomedical distinctions (e.g., placental malaria) with local understandings of *malungo* in pregnancy will be essential for community engagement with future vaccines for MiP/PM. These results provide further support for previous findings suggesting that malungo is a complex concept encompassing multiple conditions or symptoms including but not limited to malaria [15,16,22,27]. 

Education and age were not significant predictors of knowledge in any district. However, knowledge differed sharply by district, with lower knowledge scores in Mpemba compared to Madziabango and Thyolo. These findings are consistent with previous evidence from Malawi: National level survey analyses have shown that women from rural areas are significantly more likely to have less knowledge of causes and symptoms than urban women, even after adjusting for age, education, media access, and other factors [18]. Spatial modelling has also demonstrated fine scale geographical variations in malaria risk and intervention coverage across districts and eco-epidemiological zones, linked to differences in altitude, rainfall and hydrology, as well as local vector habitats [30,31]. At service level, studies have found that comprehension of ITPp and related messages differs between tertiary urban, semiurban, and rural facilities; clinic workflows and health worker practices, as well as ANC attendance also shape who is exposed to and able to internalise prevention messages [32,33,34]. In parallel, documented variation in HSA density, training, supervision, and motivation further amplifies gaps, shaping the credibility of household education. Finally, structural and logistical issues such as accessibility (distance, road, and transport) constraints reduce routine contact with facilities and can depress knowledge [35,36]. Taken together, the observed district differences are therefore plausible in the light of known rural–urban disparities in media and service access, underlying heterogeneity in malaria transmission and variation in ANC utilisation and quality across sites.

The majority of women identified bed net use as the primary preventive practice, with limited reference to antimalarial drugs or environmental controlling. These results align with national ITNs coverage, indicating that large-scale distribution campaigns have significantly increased ownership and reported usage, even though impact and coverage remain diverse across the country [37,38,39]. The low uptake of antimalarial drugs in Malawi is attributed to structural challenges such as limited or restricted access to healthcare, stockouts, HCW attitudes, and insufficient knowledge about antimalarials [40,41]. There was no significant correlation between age or education level and preventive practices; a previous Malawi study found similar results [33]. This suggests widespread ITNs and basic messaging through ANC and community campaigns can make prevention a community norm, regardless of formal education. Preventive behaviour was higher in Thyolo compared to both Madziabango and Mpemba, with no significant difference between the latter two. These differences may reflect differences in access to health promotion plans, distribution of mosquito nets, facility effectiveness and accessibility, or community engagement. Instead of assuming uniform uptake across rural areas, district-specific implementation strategies should be put in place. Neither education nor age was associated with the perceived risk of disease. Earlier work from rural Malawi showed that understandings of *malungo* risks are embedded in local illness concepts and everyday experience rather than in formal education or biomedical messaging alone [15,27,42].

By contrast, the strong district level differences point to the importance of place and local context in shaping how risk is perceived. Women in Mpemba were significantly less likely to perceive their area as high risk compared to those in Thyolo and Madziabango, despite the fact that in national context almost all Malawians are considered at risk for malaria [13,29]. This pattern is consistent with wider evidence that malaria transmission and perceived risk are highly spatially heterogeneous, reflecting local ecologies, exposure patterns and programme histories [43,44]. One possible explanation is that lower perceived risk in Mpemba reflects recent reductions in visible malaria burden, variations in vector ecology, or lower local emphasis on malaria in health communication. From a programmatic perspective, these findings suggest that risk communication efforts must be locally adapted and cannot presume that national-level “high burden” labels align with community-level perceptions of risk.

ANC nurses were the most commonly reported source, with far fewer citing HSAs, TBAs, relatives, and other informal sources. Although women valued ANC and recognised its role in maternal health counselling, evidence from Malawi shows that this does not always translate into practice: many women still initiate ANC late, usually in the second or third trimester, and may not receive consistent and adequate information during the visit [45,46]. Structural and social barriers such as long distances, limited resources, low decision-making power and negative interactions with ANC further limit the usefulness of ANC as a reliable information source [45,47]. Although survey participants denied using TBAs, this finding should be interpreted cautiously in light of Malawi’s historical policy environment. While contemporary large-scale studies do not robustly document TBA-led deliveries in Malawi following the 2007–2008 national ban on Traditional Birth Attendants (TBAs), historical and policy research suggest that TBA use was reduced but not completely eliminated, as the ban was lifted in 2010 [48,49]. In remote areas, residual and informal use may persist but go unreported because of legal, programmatic, and social pressure, as well as the protection of TBAs by community members [50,51,52]. This potential underreporting should be considered when interpreting our survey findings. Small but persistent recourse to traditional practices (e.g., *mulimbiko* from *Hippocratea parviflora*) underscores how women negotiate perceived benefits and risks when formal care is distant, under-resourced, or culturally mismatched [28,53]. Health counselling patterns varied across districts, and were also associated with age, with older women more likely to rely exclusively on biomedical counselling. Women in Thyolo and Mpemba were more likely to rely on formal health services and biomedical counselling, whereas women in Madziabango also relied on mixed or informal counselling sources. This likely reflects uneven HSA reach, patient–provider relationships, perceived value of services and facility performance, financial constraints, as well as accessibility, factors that have frequently been shown to shape the credibility and uptake of ANC advice [54,55].

Vaccine attitudes of participants show a familiar paradox: very high uptake and stated willingness alongside pervasive safety concerns. The item on vaccine harm likely captures general awareness that medical interventions may carry some risk, rather than specific safety concerns about vaccination. In addition, stated willingness to accept a hypothetical vaccine may overestimate real-world uptake, particularly in the context of interviewer-administered surveys, where acquiescence and social desirability bias may influence responses.

Around 92% of women voiced concern of potential harm from vaccines, similar to other Malawi studies that document safety narratives circulating alongside acceptance, with hesitancy shaped more by trust, rumours, and social networks than by simple pro/anti binaries [56,57]. The finding that women who worried about harm were more likely to say they would take a future malaria vaccine is not contradictory: This tension reflects a form of “anxious compliance,” where women accept vaccination despite persistent concerns, a pattern also noted in other maternal vaccine studies [58,59]. Studies show that provider recommendations and social norms can outweigh anxiety, producing “compliant but cautious” behaviour [56,60]. There is a complex interplay between trust in medical authority, cultural values regarding motherhood, and persistent safety concerns in shaping vaccine behaviours among pregnant women. This pattern may facilitate the introduction of maternal malaria vaccines, as Malawi’s prior role as an RTS,S pilot country demonstrated feasibility and strong parental demand when vaccines are offered through routine services with clear guidance.

Education and age were not significantly associated with vaccine attitudes. These findings are consistent with earlier research in Africa showing that education and demographic factors are not strong predictors of vaccine acceptance [61,62,63]. Other factors more reliably predict vaccine behaviour, including trust in health authorities, perceived vaccine safety and effectiveness, access to accurate health information, and social norms within communities [64,65,66,67]. However, district differences in attitude indices show that acceptance is locally dependent, likely due to communication, logistics, and recent service experience. RTS,S pilot implementation reviews highlight these determinants (cold-chain reliability, timely counselling, dose-schedule support) [68]. High willingness is a strong foundation, but concerns about vaccine safety, the growing number of vaccines for girls, and questions about the need for vaccines [64,69,70] must be addressed beforehand to promote community uptake through trustworthy, repeated ANC counselling, community engagement, and rapid issue management to ensure on-time, complete series when pregnancy malaria vaccines become available.

## 6. Conclusions

This survey showed high general awareness of *malungo* and strong willingness to be vaccinated against maternal malaria/malungo-in-pregnancy. There were marked district-level differences in knowledge, perceived risk, counselling patterns, and vaccine attitudes. Future MiP/PM vaccines should therefore prioritise communication that bridges biomedical concepts of PM with local illness concepts, addresses safety concerns directly, and tailors social and behavioural change strategies to district context, rather than uniform messaging to ensure that expressed willingness translates into timely uptake.

## Figures and Tables

**Table 1:** Background characteristics of participants in surveys in Thyolo and rural Blantyre, Malawi.

Variable	Characteristics	N (of Total)	%
Gender	Female	303	99.3
	Missing data	2	0.7
Age	<20	60	19.7
	20–29	172	56.4
	30–39	67	22.0
	40–49	6	2.0
	Median age = 24		
	Mean age = 25.3		
Education	None	8	2.6
	Primary	191	62.6
	Secondary	97	31.8
	Higher	1	0.3
	Missing data	8	2.6
Location	Mpemba	106	34.8
	Thyolo	101	33.1
	Madziabango	98	32.1

**Table 2:** Adjusted associations for predictors of above-median malaria knowledge. Adjusted odds ratios (aORs) with 95% confidence intervals applied to pairwise district comparisons, education, and age groups. Outcome: above median knowledge index ≥ 0.69.

Variable	aOR	95% CI	*p*-Value
Education (higher vs. lower)	1.83	1.09–3.11	0.024
Age (≥25 vs. <25 years)	0.87	0.54–1.4	0.561
District: Madziabango vs. Thyolo	0.85	0.48–1.5	0.572
District: Mpemba vs. Thyolo	0.23	0.12–0.41	<0.001
District: Mpemba vs. Madziabango	0.27	0.14–0.49	<0.001

**Table 3:** Knowledge of *malungo* prevention methods among women of reproductive age.

Do You Know How to Prevent *Malungo*	Participants	Percentage (%)
Sleeping under mosquito net	160	52.5%
Sleeping under mosquito net & filling the stagnant water bodies	19	6.2%
Sleeping under mosquito net and clearing surrounding	17	5.6%
Sleeping under mosquito net and burning coil	19	6.2%
Sleeping under mosquito net and taking antimalarial drugs	6	2.0%
Other option (this includes burning animal dung& herbs, burning coils combined with either taking malaria drugs or using mosquito repellents, and environmental measures such as clearing surroundings, filling stagnant water bodies, constructing waterways, and taking care of utensils to reduce breeding grounds).	38	12.5%
No as response	24	7.9%
Yes (without explanation)	16	5.2%
**Do you use any malaria prevention methods right now?**	**Count**	**% of all responses**
Net only	202	66.2%
Net + Antimalarials	54	17.7%
Net + Insecticides	13	4.3%
Net + Repellents	6	2.0%
Net + other options (Participants mentioned sleeping net with more than one options + clearing surroundings/herbal medicine/other ways/sanitation/spraying insecticides + coil + doom + using mosquito repellent/taking malaria drugs, skin care products, spraying pesticides.)	17	5.6%
Other options	6	2.0%
Missing data	7	2.3%

**Table 4:** Adjusted associations for predictors of malaria prevention behaviour. Adjusted odds ratios (aORs) with 95% confidence intervals applied to pairwise district comparisons, education, and age groups. Outcome: above or equal median behaviour index ≥ 0.92.

Variable	aOR	95% CI	*p*-Value
Education (higher vs. lower)	1.57	0.88–2.81	0.126
Age (≥25 vs. <25 years)	1.06	0.61–1.85	0.831
District: Madziabango vs. Thyolo	0.26	0.13–0.5	<0.001
District: Mpemba vs. Thyolo	0.26	0.13–0.49	<0.001
District: Mpemba vs. Madziabango	1	0.47–2.17	0.997

**Table 5:** Adjusted associations for predictors of high perceived risk awareness. Adjusted odds ratios (aORs) with 95% confidence intervals applied to pairwise district comparisons, education, and age groups. Outcome: above or equal median risk index ≥ 0.33.

Variable	aOR	95% CI	*p*-Value
Education (higher vs. lower)	0.94	0.56–1.57	0.817
Age (≥25 vs. <25 years)	1.12	0.7–1.79	0.643
District: Madziabango vs. Thyolo	0.81	0.46–1.42	0.455
District: Mpemba vs. Thyolo	0.31	0.17–0.56	<0.001
District: Mpemba vs. Madziabango	0.39	0.21–0.69	0.002

**Table 6:** Adjusted associations for predictors of biomedical health counselling behaviour. Adjusted odds ratios (aORs) with 95% confidence intervals applied to pairwise district comparisons, education, and age groups. Outcome: Exclusively biomedical health counselling index = 1.

Variable	aOR	95% CI	*p*-Value
Education (higher vs. lower)	0.98	0.55–1.78	0.958
Age (≥25 vs. <25 years)	2.1	1.22–3.69	0.009
District: Madziabango vs. Thyolo	0.4	0.21–0.76	0.006
District: Mpemba vs. Thyolo	1.14	0.56–2.33	0.716
District: Mpemba vs. Madziabango	2.84	1.49–5.58	0.002

**Table 7:** Comparison between respondents who believed in potential harmful effects of vaccines and those who did not. Respondents who replied, “Don’t know”, or for whom data was missing on this item were excluded for this comparison. Most common answer in each group is highlighted in bold. The question “Do you think vaccines carry the potential of harm for your baby?” was answered by 2.0% (6 of 305) with “I don’t know”, and by 1.0% (3 of 305) with either “not applicable (NA)” or with no answer. In this table, only “yes” responses (92.1%, *n* = 281) and “no” responses (4.9%, *n* = 15) were included to understand how they correlate with answers to vaccine uptake.

	Respondents Who Answered “Yes” to Potential Harm of Vaccines (*n* = 281)				Respondents Who Answered “No” to Potential Harm of Vaccines (*n* = 15)			
Survey Question	Yes	No	I Do Not Know	NA, or No Answer	Yes	No	I Do not Know	NA, or No Answer
“Have you been vaccinated for tetanus in your last/present pregnancy?”	261 (92.6%)	18 (6.4%)	-	2 (1.1%)	14 (93.3%)	1 (6.7%)	-	-
“Are you going to have your baby vaccinated after birth?”	48 (17%)	8 (2.6%)	-	226 (73.9%)	6 (40%)	9 (60%)	-	-
“Have you heard about *malungo* vaccines before?”	168 (59.6%)	112 (40.1%)	1 (0.4%)	-	4 (26.7%)	11 (73.3%)	-	-
“If a *malungo* vaccine becomes available in the future and is recommended for pregnant women, would you get vaccinated?”	276 (97.2%)	3 (1.1%)	2 (0.7%)	-	13 (86.7%)	2 (13.3%)	-	-
“If available, would you advise your daughter to get a PM vaccine before her pregnancy?”	268 (95.4%)	10 (3.5%)	1 (0.4%)	2 (0.7%)	12 (80%)	3 (20%)	-	-

**Table 8:** Adjusted associations for predictors of positive vaccine attitudes. Adjusted odds ratios (aORs) with 95% confidence intervals applied to pairwise district comparisons, education, and age groups. Outcome: above or equal to median vaccine attitude index ≥ 0.8.

Variable	aOR	95% CI	*p*-Value
Education (higher vs. lower)	1.23	0.7–2.19	0.482
Age (≥25 vs. <25 years)	1.47	0.88–2.5	0.146
District: Madziabango vs. Thyolo	0.53	0.28–1.01	0.057
District: Mpemba vs. Thyolo	0.79	0.41–1.53	0.495
District: Mpemba vs. Madziabango	1.49	0.8–2.77	0.209

## Data Availability

The datasets generated and analysed during this current study are available from the corresponding author upon reasonable request. Data were anonymized prior to analysis.

## Supplementary Materials

The following supporting information can be downloaded at: https://www.mdpi.com/article/10.3390/vaccines14040316/s1, Table S1. Knowledge about cause of malungo in relation with education among women of reproductive age in Thyolo and Blantyre (rural), Malawi; Table S2. Construction of composite indices for knowledge, behaviour, perceived risk, health counselling and vaccine attitude. If a respondent’s answer to at least one question within an index was missing, no index for that category was calculated for that respondent. If answers to other categories were complete, the respondent was however included for those categories and indexes were calculated. Table S3: Number of missing replies or ‘na’ for each item. For most questions, missing data was below 3%. For questions with a higher percentage of missing answers, see the comment column. Some answers could not be enumerated for the index, for example when participants answered, “Do you know how to prevent malungo?” with “yes”, without explicitly mentioning a method.

## Abbreviations

The following abbreviations are used in this manuscript:

ACT	Artemisinin-based combination therapy
AIDS	Acquired Immune Deficiency Disorder
ANC	Antenatal Clinic
BI	Behavioural Index
CI	Confidence intervals
CM	Congenital malaria
CSA	Chondroitin Sulphate A
DGIS	Directorate-General for International Cooperation
FGD	Focus group discussion
HC	Health Clinic
HCI	Health Counselling Index
HSA	Health surveillance assistant
IDIs	In-depth interviews
IPTp	Intermittent preventive treatment
IRS	Indoor residual spray
ITNs	Insecticide-treated nets
KAP	Knowledge, attitude, practices
KI	knowledge Index
MBS	Malawi Behaviour Survey
MIP	Malaria in pregnancy
MMIS	Malawi Malaria Indicator Survey
MUSTREC	Malawi University of Science and Technology Research Ethics Committee
NA	Not applicable
NMCP	National Malaria Control Program
NSO	National Statistical Office
OR	Odds ratio
PAM	Pregnancy-associated malaria
PM	Placental Malaria
RI	Risk Index
SAGE	Strategic Advisory Group of Experts on Immunization
SP	Sulfadoxine-pyrimethamine
SSA	Sub-Saharan Africa
TBA	Traditional Birth attendants
TH	Traditional Healers
VI	Vaccine Attitude index
VIF	Variation inflation factor
WHO	World Health Organization
WMR	World Malaria Report
