# Supplementary material for: Assessing Readiness for Future Maternal Malaria Vaccines: Knowledge, Practices, and Vaccine Attitudes Among Women of Reproductive Age in Malawi
Source: Vaccines (Basel). 2026 Mar 31;14(4):316. doi: 10.3390/vaccines14040316 (PMC13120181; doi:10.3390/vaccines14040316)
Supplement: Supplementary file 1 [file vaccines-14-00316-s001.zip › vaccines-4188982-supplementary.pdf]

Table S1. Knowledge about cause of malungo in relation with education among women of reproductive age in Thyolo and Blantyre (rural), Malawi

**Do you know what causes *malungo*?**	**Primary education**	**Secondary Education**	**Missing data**	**Not attended**	**Total**	**%**
**Sole cause**					**189**	**61.6**
mosquitoes	120	62	2	5	189	
**I don't know/Unclear answers**					**53**	**17.3**
No	26	6	2	3	36	
Yes	9	4	2		15	
**Mixed (mosquito + lack of prevention methods)**					**31**	**10.1**
mosquito & not sleeping under net	13	7			20	
mosquito & keeping stagnant waters	1	2	1		4	
mosquitoes & lack of cleanliness	1				1	
mosquitoes & coldness	1				1	
mosquito & cough & flu	1				1	
mosquito & shivering	1				1	
mosquito & not taking malungo drugs		1			1	
mosquitoes & keeping stagnant waters & lack of cleanliness		1			1	
**Not following prevention methods/Lack of hygiene / surroundings**					**32**	**10.4**
not sleeping under net	10	10	1		21	
not sleeping under net & keeping stagnant waters	3	3			6	
not sleeping under net & not clearing the surrounding/cleanliness	2	1			3	
keeping stagnant waters & not clearing the surroundings /cleanliness	1	1			2	
**Symptoms mentioned as cause**					**2**	**0.65**
shivering	1				1	
feeling cold & fever	1				1	

**Grand Total**	**191**	**97**	**8**	**8**	**305**

Table S2. Construction of composite indices for knowledge, behaviour, perceived risk, health counselling and vaccine attitude. If a respondent's answer to at least one question within an index was missing, no index for that category was calculated for that respondent. If answers to other categories were complete, the respondent was however included for those categories and indexes were calculated.

	**Question**	**Response Coding**	**Threshold**
**Knowledge Index (KI)**	K1. Have you heard of a disease called *'malungo'*?	1= yes, 0 = no	ki calculated as the mean of K1, K2 and K4 (excluding "–" = missing/NA). Range 0–1.<br>**Categories:**<br>poor knowledge: ki < 0.5<br>medium knowledge: 0.5 - 0.75 ki<br>good knowledge: 0.75 < ki
	K2. Do you know how to prevent *malungo*? Please explain.	1 = sleeping under nets + one or more good supplementary method/s*<br>0.75 = sleeping under nets and no other method<br>0.5 = sleeping under nets + ineffective methods**<br>0.5 = only supplementary methods<br>0.25 = supplementary methods + ineffective methods<br>0 = only ineffective methods or none	Contributes to ki as above. See Notes below for what were considered supplementary and what were considered ineffective methods.
	**K3. Have you heard of *malungo* vaccines before?**	**1= yes; 0= no; 0.5 = I don't know; '-'= missing/NA**	**Not included in ki. Used descriptively and qualitatively to explore awareness of malaria vaccines.**
	K4. Do you know what causes *malungo*? Please explain.	1= mosquitoes only; 0.5 = mosquitoes + other causes; 0= other/none	Contributes to ki as above. Some participants mentioned lack of prevention techniques such as not using bed nets as the cause for malungo, or confused symptoms like as shivering as the cause of the disease. All answers that did not mention mosquitoes were rated as 0.

**Prevention behaviour Index (BI)**	B1. Have you used any *malungo* prevention methods in the past?	1= yes; 0 = no; 0.5 = I don't know; '-' = missing data/NA	bi calculated as the mean of B1–B3 (excluding "–"). Range 0–1. Categories: poor behaviour bi < 0.5; medium behaviour bi 0.5 - 0.75; good behaviour bi > 0.75.
	B2. Do you use any *malungo* prevention methods right now?	1= sleeping under nets + ≥1 "good" supplementary method/s*; 0.75 = sleeping under nets only; 0.75 = sleeping under nets + ineffective methods**; 0.5 = only supplementary methods; 0.25 = supplementary methods + ineffective; 0 = only ineffective methods or none	Contributes to bi as above. For scoring of what counts as supplementary methods and ineffective methods see Notes. Ranking is slightly different from K2: Sleeping under a net as the only method is equally as effective as protection as sleeping under a net combined with an ineffective method – they are therefore both scored as 0.75.
	B3. Have you ever taken, or will you take medication if you get *malungo* in pregnancy? If no, please explain why not.	1= Yes; 0 = No; '-' = missing data/NA	Contributes to bi as above
**Risk awareness Index (RI)**	R1. Do you have a high risk of *malungo* transmission in your area?	1= Yes; 0 = No; 0.5 = I don't know; '-' = missing data/NA.	ri calculated as the mean of R1–R3 (excluding "–"). Range 0–1. Categories: low perceived risk ri < 0.5; medium 0.5 - 0.75; high > 0.75.
	R2. Did you have *malungo* in last pregnancy?	1= Yes; 0 = No; '-' = missing data/NA	Contributes to ri
	R3. Did you have *malungo* in any previous pregnancy?	1= Yes; 0 = no; '-' = missing data/NA	Contributes to ri
**Health Counselling (HC)**	H1. Whom do you take general health advice from?	1= ANC nurses and/or HSA; 0.5 = ANC nurses/HSA + TBA/relatives, friends, / community; 0 = TBA/ parents, relatives, community '-'= missing data/NA	Responses grouped into two categories: exclusively biomedical (ANC/HSAs only); not exclusively biomedical
	H2. Whom do you take advice from to take any medication during pregnancy?	1 = ANC nurses and/or HSA; 0.5 = ANC nurses/HAS + TBA/relatives/ friends/parents/pharmacies; 0 = only parents/	Used together with H1 to describe main sources of medication advice (exclusively biomedical, not exclusively biomedical).

		relatives/"I don't take drugs"/"people I live with"/grocery store; ('-')= missing data /NA	
	**H3. Do you visit Traditional Birth Attendants (TBAs), Traditional Healers (THs), or Antenatal Clinics (ANC) during your pregnancy?**	**Multiple heterogenous responses**	**Not scored as an index item. Analysed qualitatively to explore how women perceive, justify or reject traditional versus biomedical care.**
	H4. Was the tetanus vaccine recommended in the present pregnancy?	1= Yes; 0 = No; '-' = missing data/NA	Used descriptively as indicator of exposure to ANC vaccination counselling
**Vaccine attitude Index (VI)**	V1. Have you ever been vaccinated?	1= Yes; 0 = No; '-' = missing data /NA	vi calculated as the mean of V1, V2, V3 (reverse-coded), V5 and V6 (excluding "–"). Range 0–1. Categories: vaccine sceptical: vi < 0.5; vaccine ambivalent: 0.5 - 0.75; vaccine open: > 0.75.
	V2. Have you been vaccinated for tetanus in the last pregnancy?	1= Yes; 0 = No; '-' = missing data/NA	Contributes to vi as above.
	V3. Do you think that vaccines carry the possibility of harm for your baby?	1= Yes; 0 = No; 0.5 = I don't know; '-' = missing data/NA	Reverse-coded when constructing vi (i.e. higher values after recoding indicate lower perceived harm / more positive attitude).
	**V4. Are you going to have your baby vaccinated after birth?**	**99% did not answer the question only 1 said yes**	**Not included in vi due to near-complete non-response; reported descriptively only.**
	V5. If a *malungo* vaccine becomes available in the future and is recommended for pregnant women, would you get vaccinated? If not, please explain why.	1= Yes; 0 = No; '-' = missing data	Contributes to vi as indicator of maternal malaria vaccine acceptance.
	V6. Would you advise your daughter to get a "*malungo* in pregnancy vaccine" before her pregnancy?	1= Yes; 0 = No; 0.5= I don't know; '-' = missing data	Contributes to vi as indicator of willingness to recommend maternal malaria vaccination for daughters.

Notes: *Supplementary methods include insecticides, antimalarial drugs, using mosquito repellent skin care products, coils, and filling stagnant water bodies. Supplementary methods are most effective when paired with bed nets as a preventive measure.

**Methods mentioned by participants considered ineffective: General hygiene, cleaning the house, beating around bushes, food hygiene, herbal medicine.

Table S3. Number of missing replies or 'na' for each item. For most questions, missing data was below 3%. For questions with a higher percentage of missing answers, see the comment column. Some answers could not be enumerated for the index, for example when participants answered, "Do you know how to prevent malungo?" with "yes", without explicitly mentioning a method.

	Question	Missing	Answer cannot be scored for index	Missing and unscorable in %	Comment
Demographics	Age	-	-	-	
	sex	2	-	0.7%	
	education	8	-	2.6%	
	district	-	-		
	K1. Have you heard of a disease called '*malungo*'?	-	-	-	
	K2. Do you know how to prevent *malungo*? Please explain.	-	16	-	Unscorable were "yes" answers without explanation
	K3. Have you heard about *malungo* vaccines before?	2	-	0.3%	
	K4. Do you know what causes *malungo*? Please explain.	-	15		Unscorable were "yes" answers without explanation
	B1. Have you used any *malungo* prevention methods in the past?	2	-	0.3%	
	B2. Do you use any *malungo* prevention methods right now?	6		2%	
	B3. Have you ever taken, or will you take medication if you get *malungo* in pregnancy? If no, please explain why not.	3		1%	
	R1. Do you have a high risk of *malungo* transmission in your area?	7	-	2%	
	**R2. Did you have *malungo* in last pregnancy?**	**18**		**5.9%**	

	R3. Did you have *malungo* in any previous pregnancy?	1		0.3%	
	H1. Whom do you take general health advice from?	1		0.3%	
	H2. Whom do you take advice from to take any medication during pregnancy?	2		0.7%	
	**H3. Do you visit Traditional Birth Attendants (TBAs), Traditional Healers (THs), or Antenatal Clinics (ANC) during your pregnancy?**	**28**		**9.1%**	For this question there were many heterogenous responses, so it was analysed in a qualitative manner
	H4. Was the tetanus vaccine recommended in the present pregnancy?	4		1.3%	
	V1. Have you ever been vaccinated?	1		0.3%	
	V2. Have you been vaccinated for tetanus in the last pregnancy?	5		1.6%	
	V3. Do you think that vaccines carry the possibility of harm for your baby?	3		1%	
	**V4. Are you going to have your baby vaccinated after birth?**	**228**		**74.3%**	This question was not included in the vaccination index vi due to the high number of non-response.
	V5. If a *malungo* vaccine becomes available in the future and is recommended for pregnant women, would you get vaccinated? If not, please explain why.	1		0.3%	
	V6. Would you advise your daughter to get a "*malungo* in pregnancy vaccine" before her pregnancy?	3		1%	
